# Identifying care problem clusters and core care problems of older adults with dementia for caregivers: a network analysis

**DOI:** 10.3389/fpubh.2023.1195637

**Published:** 2023-08-10

**Authors:** Minmin Leng, Shuyu Han, Yue Sun, Zheng Zhu, Yajie Zhao, Yizhu Zhang, Xianxia Yang, Zhiwen Wang

**Affiliations:** ^1^Department of Nursing, Shandong Provincial Hospital Affiliated to Shandong First Medical University, Jinan, China; ^2^School of Nursing, Peking University, Beijing, China; ^3^School of Nursing, Fudan University, Shanghai, China; ^4^Department of Cardiology, Beijing Hospital, National Center of Gerontology, Institute of Geriatric Medicine, Chinese Academy of Medical Sciences, Beijing, China; ^5^School of Public Health, Wuhan University, Wuhan, China

**Keywords:** dementia, caregivers, care problem, cluster, symptom network, network analysis

## Abstract

**Background:**

A shift in research interest from separate care problem to care problem clusters among caregivers of people living with dementia may contribute to a better understanding of dementia care. However, the care problems network among caregivers of people living with dementia are still unknown. This study aimed to identify care problem clusters and core care problems, and explore demographic variables associated with these care problem clusters among caregivers of people living with dementia.

**Methods:**

Participants were recruited through memory clinics and WeChat groups. The principal component analysis was applied to identify care problem clusters. The network analysis was conducted to describe the relationships among care problems and clusters. Multiple linear models were used to explore the associated factors for the occurrence of the overall care problems and top three central care problem clusters.

**Results:**

A total of 1,012 carer-patient pairs were included in the analysis. Nine care problem clusters were identified. In the entire care problem network, “deterioration in activities of daily living” was the most core care problem cluster across the three centrality indices, followed by “verbal and nonverbal aggression” and “loss of activities of daily living.” Variables including marital status, years of dementia diagnosis, number of dementia medication type, and caregiver’s educational attainment were associated with the prevalence of these three care problem clusters.

**Conclusion:**

Our study suggests that there is a need to evaluate care problem clusters for the improvement of care problem management among people living with dementia. It is particularly important to include assessment and treatment of core care problem as an essential component of the dementia care.

## Introduction

Dementia affects more than 55 million people worldwide, with a new case of dementia occurring around the world every 3 s; this number is expected to increase to 78 million by 2030 and 139 million by 2050 (1). China is one of the countries with the fastest growth in the older population. It is estimated that 15.07 million people aged 60 years or older in China living with dementia (2), accounting for about 25% of the global dementia population (3). The number of dementia cases in China is expected to reach 45.54 million by 2050 (4). The incidence, morbidity and mortality rates of dementia have steadily increased to make it presently the fifth leading cause of death among urban and rural residents in China and magnify the resulting burdens on individuals, families and society. The lack of effective treatment for dementia promotes the transition from disease treatment to health maintenance for this expanding population with dementia. In the long-term health maintenance, caregivers are faced with diversified and complicated care problems. Care problems refer to various difficulties that caregivers encounter in the process of taking care of people living with dementia (5), involving various aspects of managing daily living (6, 7), behavioral and psychological symptoms (8, 9), and safety risks (10, 11). Compared with activities of daily living (ADL) or behavioral and psychological symptoms of dementia (BPSD), care problems include a wider range and more detailed items. Effective management of these care problems is crucial because they are associated with accelerated cognitive decline (12), poorer quality of life (13), impaired daily functioning (14), and increased risk of institutionalization (15). However, the variety of care problems makes it difficult for caregivers to cover all aspects. It is of great significance to reduce the dimension of care problems and identify the core care problems.

Exploring symptom clusters is a classic analytical paradigm for reducing the dimension to simplify complex scenarios in real-world clinical practice. The defining characteristics of a symptom cluster are described as two or more symptoms co-occurring, which may have shared underlying mechanisms or shared outcomes (16–18). In the field of dementia care, most current studies have focused on isolated care problems and thus may fail to represent the real-world situation where caregivers of people living with dementia usually have experienced more than one care problem (19, 20). Symptom clusters provide an idea for reducing the dimension of care problems for caregivers of people living with dementia. A recent study (21) conducted cluster analysis of care problems based on the minimum spanning tree algorithm on caregivers of people living with dementia. But the minimum spanning tree can only focus on the strength between care problems, and the association network among care problem clusters cannot be identified. There are a variety of analytical approaches to identify symptom clusters (22, 23), among which network analysis is a novel statistical approach that models the relationship between symptomatic constructs at the component level. The network nodes represent variables and network edges represent relationships between variables. Network analysis has many advantages in the identification of symptom clusters: (1) can not only focus on the strength, but also on the betweenness and closeness between symptoms; (2) can not only identify symptom clusters, but also the association network and centrality indices among symptom clusters; and (3) can focus on the microlevel interactions among symptoms. Currently, network analysis has been applied to patients with mental illness (24–26), cancers (27, 28), and acquired immune deficiency syndrome (29–31).

With the application of network analysis in symptom cluster identification, symptom network paradigm was proposed (32). Symptom networks not only have the function of reducing dimensionality similar to symptom clusters, but also can guide researchers and healthcare providers to develop precise personalized health interventions. The main functions of the symptom network paradigm include clustering symptoms, identifying core symptoms, determining the density of symptom network, and focusing on micro-level interactions among symptoms (32–34). However, the care problems network among caregivers of people living with dementia are still unknown. Under the guidance of symptom network paradigm, this study aimed to achieve the following goals through network analysis: (1) cluster care problems among caregivers of people living with dementia; (2) identify core care problems among caregivers of people living with dementia; and (3) explore demographic and health-related factors associated with these care problem clusters.

## Methods

### Setting and study participants

This study was approved by the Peking University Biomedical Ethics Committee (IRB00001052-21095). We recruited dementia caregivers in memory clinics in Beijing and WeChat groups established by memory clinic physicians in Beijing, Tianjin, Hangzhou, and Guangzhou between September 2019 and October 2021. Data collectors in each study setting received training and collected data through paper-based questionnaires or online questionnaires. They explained the study objectives and procedures to the participants and obtained the informed consent from them. Participants usually spent 15–20 min completing the questionnaires. Participants were eligible if they: (1) aged 18 years or older; (2) had care recipients who were diagnosed with dementia; (3) undertook the main care task for people with dementia for more than 3 months; (4) were able to provide people with dementia’s personal information; (5) signed the informed consent form.

### Measures

#### Demographic and clinical characteristics

A standard questionnaire was used to collect data of people with dementia’s demographic and clinical characteristics, and caregivers’ demographic characteristics. People with dementia’s demographic variables included gender, age, educational attainment, and marital status. Their clinical variables included diagnosis, years of dementia diagnosis, number of dementia medication type, and number of chronic diseases. Data of gender, age, educational attainment, relationship with care recipients, and employment status were collected to describe caregivers’ demographic characteristics.

#### Care problem

A dementia caregiver’s care problem checklist that designed by our research team was applied to assess participant’s care problems (5). Our research team combined the specific performance of people living with dementia, based on literature review, and conducted caregiving experience interviews with dementia caregivers to form the first draft of a dementia caregiver’s care problem checklist. Then, 32 dementia care experts were selected for two rounds of expert letter consultation. The importance of each item was rated, and each item could be modified and supplemented to form the final draft of the dementia caregiver’s care problem checklist. This checklist included three dimensions: daily living care problems (30 items), behavioral and psychological problems (15 items), and safety risk problems (13 items). Each item responded 0 (no) or 1 (yes). Daily living care problems and behavioral and psychological problems had a recall period of 2 weeks. For safety risk problems, participants were asked to report if they had experienced these care problems in the past 3 months. This assessment tool showed satisfying content validity (CVI = 0.879) and internal consistency (Cronbach’s *α* = 0.857). Detailed information of dementia caregiver’s care problem checklist is available in the Supplementary material.

### Data analysis

We applied SPSS 24.0 and Python 3.6.0 for statistical analysis. The mean and standard deviation (S.D.) was estimated for continuous variables and the frequencies and percentages for categorical variables. The principal component analysis (PCA) using the orthogonal transformation (varimax rotation) was performed to identify care problem clusters. Kaiser–Meyer–Olkin test was first conducted to judge if the dataset was suitable for factor analysis. Factors with eigen values greater than 1.0 were included. The number of factors was also determined by a scree plot. We defined factor loading that greater than 0.40 were eligible for clusters. The results of care problem clusters were discussed about clinical relevance within our research group.

The network analysis was performed to describe the relationships among care problems and clusters. The Spearman correlation was applied to estimate the correlation relationships between the nodes in the networks; the Fruchterman–Reingold algorithm was applied to place nodes with the strongest correlations at the center of the network (35). Centrality indices, including strength, closeness, and betweenness were used to identify the most central care problem and clusters in the networks (32). All the centrality indices were standardized (reporting *r* between 0 and 1) to make all the nodes more comparable.

Multiple linear models were used to explore factors associated with the overall care problem and top three central care problem clusters in the cluster network. Independent variables included demographic and clinical variables of people with dementia, and demographic variables of caregivers. Multicollinearity is diagnosed when the variance inflation factor (VIF) is higher than 5 (36). A *p*-value <0.05 was considered to be statistically significant for all the data analysis.

## Results

### Descriptive analysis

A total of 1,105 carer-patient pairs participated in our study, 93 (8.4%) of were excluded because of missing data or invalid data logic. Participants’ and their care recipients’ characteristics are shown in Table 1. The majority of people with dementia were female (52.6%), married (61.4%), and diagnosed with Alzheimer’s disease (77.1%). Most of them did not have a high education level, which only 12.5% of them had a bachelor’s degree or above. Their average age was 75.43 years old. The average years of dementia diagnosis was 3.89 years. More than 90% of the people with dementia took more than one type dementia medication, and approximately 75% of the them had at least one kind of chronic disease. The majority of participants were female (66.0%) and were offspring of the people with dementia (61.0%). Their average age was about 50 years old. More than half of the participants had a bachelor’s degree or above (55.5%), and had a full-time job or part-time job (55.6%).

**Table 1:** Participant characteristics (*N* = 1,012).

Characteristics	*N* (%), *M* ± SD
**People with dementia**	
Gender	
Male	480 (47.4)
Female	532 (52.6)
Age	75.43 ± 10.53
Educational attainment	
Illiteracy	153 (15.1)
Primary school	313 (30.9)
Middle school	253 (25.0)
Senior high school	166 (16.4)
Bachelor’s or above	127 (12.5)
Marital status	
Single (including widowed and divorced)	389 (38.4)
Married	623 (61.6)
Diagnosis	
Alzheimer’s disease	780 (77.1)
Vascular dementia	76 (7.5)
Mixed dementia	99 (9.8)
Other	57 (5.6)
Years of dementia diagnosis	3.89 ± 3.20
Number of dementia medication type	
0	99 (9.8)
1	550 (54.3)
2	283 (28.0)
≥3	80 (7.9)
Number of chronic disease	
0	255 (25.2)
1	328 (32.4)
2	251 (24.8)
≥3	178 (17.7)
**Caregivers**	
Gender	
Male	344 (34.0)
Female	668 (66.0)
Age	50.26 ± 13.24
Educational attainment	
Primary school	42 (4.2)
Middle school	166 (16.4)
Senior high school	242 (23.9)
Bachelor’s or above	562 (55.5)
Relationship with care recipients	
Spouse	202 (20.0)
Daughter/son	617 (61.0)
Daughter/son-in-law	79 (7.8)
Other	114 (11.3)
Employment status	
Full-time job	481 (47.5)
Part-time job	82 (8.1)
Retired	323 (31.9)
Unemployed	126 (12.5)

### Factor analysis

The Kaiser–Meyer–Olkin value in our study was 0.845, and Bartlett’s test of sphericity was significant (*p* < 0.01), which indicated that our dataset was suitable for PCA. As presented in Table 2, nine care problem clusters were identified. Eleven care problems, including eating or drinking inappropriate substances, performing repeated action, forgetting that he/she had eaten and wanted to eat again, making verbal or physical sexual advances, dysphagia, day and night reversed, getting up multiple times during the night, having difficulty in falling asleep, feeding through a nasogastric tube, apathy, and constipation, had low loading on all factors. The most common care problem cluster was Cluster A (80.9%), followed by Cluster B (77.5%), and Cluster D (49.5%).

**Table 2:** Characteristics of care problem clusters.

Care problems	Factor loading	Number of participants (%)
**Cluster A (Deterioration in activities of daily living)**		819 (80.9)
Do not know how to dress in order	0.703	
Forgetting steps to wash or brush	0.702	
Do not know how to clean themselves after using the toilet	0.671	
Cannot find the toilet on his/her own	0.613	
Do not know how to choose food	0.596	
Cannot choose clothes that suit the season	0.541	
Do not know how to use the toilet	0.538	
Having difficulty in expressing his/her own thoughts clearly	0.519	
Urinating and defecating in inappropriate places	0.519	
Inappropriate dressing or disrobing	0.514	
Having difficulty in understanding what others are saying	0.470	
Do not know how to use tableware properly	0.468	
**Cluster B (Paraphasia and psychosis)**		784 (77.5)
Complaining	0.696	
Constantly requesting help or attention	0.668	
Saying the same thing or asking the same question repeatedly	0.666	
Delusion	0.585	
Hallucination	0.568	
Hiding valuable things, or hoarding worthless things	0.423	
**Cluster C (Verbal and physical aggression)**		481 (47.5)
Screaming	0.685	
Hitting, kicking, pushing, or biting others	0.683	
Throwing things, tearing things, or destroying property	0.567	
Making strange noises—such as laughs, crying	0.514	
Cursing or verbal aggression	0.491	
**Cluster D (Rejection of care)**		501 (49.5)
Refusing to take a bath	0.639	
Refusing to wear clothes	0.607	
Refusing to freshen up	0.536	
Wearing the same clothes and refusing to change	0.535	
Refusing to eat or refusing to be fed	0.480	
Aggressive behavior when assisting in bathing	0.476	
**Cluster E (Safety risks associated with swallowing and walking ability)**		433 (42.7)
Choking on food	0.660	
Irritating cough	0.628	
Dysphagia	0.530	
Falling out of bed	0.521	
Falls	0.402	
**Cluster F (Walking function disorder)**		402 (39.7)
Sneaking out	0.706	
Pacing and aimless wandering	0.605	
Getting lost	0.553	
**Cluster G (Loss of activities of daily living)**		397 (39.2)
Losing the ability to communicate and can only repeat simple words	0.584	
Bedridden	0.534	
Urinary and fecal incontinence	0.465	
**Cluster H (Bedridden related complications)**		96 (9.5)
Pneumonia	0.743	
Infection	0.684	
Pressure injury	0.410	
**Cluster I (Safety risks related to accidental injury)**		140 (13.8)
Accidental aspiration	0.627	
Self-injury	0.536	
Eating or drinking inappropriate substances	0.522	
Hurting others	0.462	

### Network analysis

**Figure 1:**
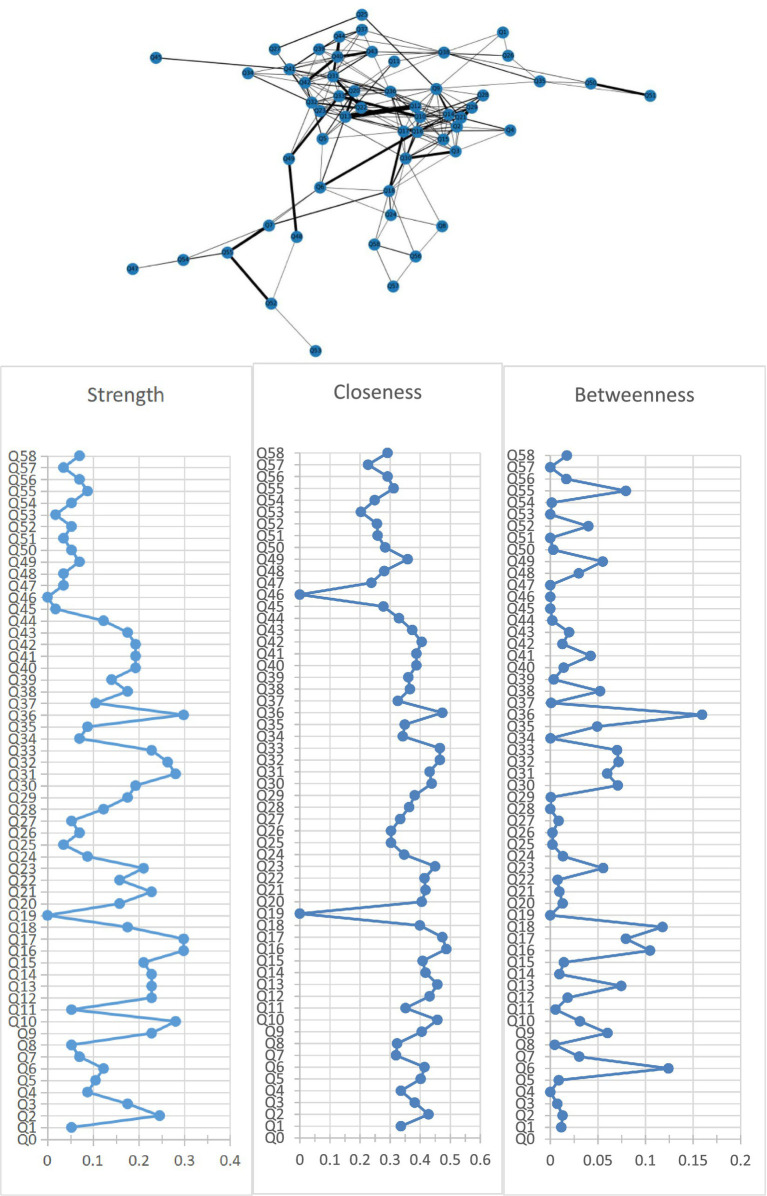
Network of care problems and centrality indices. Q1. Forgetting that he/she had eaten and wanted to eat again; Q2. Do not know how to choose food. Q3. Do not know how to use tableware properly; Q4. Eating or drinking inappropriate substances; Q5. Refusing to eat or refusing to be fed; Q6. Difficulty chewing; Q7. Dysphagia; Q8. Feeding through a nasogastric tube; Q9. Cannot choose clothes that suit the season; Q10. Do not know how to dress in order; Q11. Wearing the same clothes and refusing to change; Q12. Inappropriate dressing or disrobing; Q13. Refusing to wear clothes; Q14. Cannot find the toilet on his/her own; Q15. Do not know how to use the toilet; Q16. Do not know how to clean themselves after using the toilet; Q17. Urinating and defecating in inappropriate places; Q18. Urinary and fecal incontinence; Q19. Constipation; Q20. Refusing to take a bath; Q21. Forgetting steps to wash or brush; Q22. Refusing to freshen up; Q23. Aggressive behavior when assisting in bathing; Q24. Bedridden; Q25. Having difficulty in falling asleep; Q26. Getting up multiple times during the night; Q27. Day and night reversed; Q28. Having difficulty in understanding what others are saying; Q29. Having difficulty in expressing his/her own thoughts clearly; Q30. Losing the ability to communicate and can only repeat simple words; Q31. Cursing or verbal aggression; Q32. Hitting, kicking, pushing, or biting others; Q33. Throwing things, tearing things, or destroying property; Q34. Making verbal or physical sexual advances; Q35. Pacing and aimless wandering; Q36. Performing repeated action; Q37. Saying the same thing or asking the same question repeatedly; Q38. Hiding valuable things, or hoarding worthless things; Q39. Constantly requesting help or attention; Q40. Complaining; Q41. Making strange noises—such as laughs, crying; Q42. Screaming; Q43. Hallucination; Q44. Delusion; Q45. Apathy; Q46. Falls; Q47. Falling out of bed; Q48. Self-injury; Q49. Hurting others; Q50. Sneaking out; Q51. Getting lost; Q52. Accidental aspiration; Q53. Eating or drinking inappropriate substances; Q54. Irritating cough; Q55. Choking on food; Q56. Pneumonia; Q57. Infection; Q58. Pressure injury.

Figure 1 shows the association network and centrality indices among 58 care problems. The five strongest edges were between “cannot choose clothes that suit the season” and “forgetting steps to wash or brush” (*r* = 0.52), “hiding valuable things, or hoarding worthless things” and “constantly requesting help or attention” (*r* = 0.48), “hitting, kicking, pushing, or biting others” and “throwing things, tearing things, or destroying property” (*r* = 0.48), “hitting, kicking, pushing, or biting others” and “making strange noises–such as laughs, crying” (*r* = 0.47), and “do not know how to dress in order” and “do not know how to clean themselves after using the toilet” (*r* = 0.47). In the entire network, “performing repeated action” (*r*_S_ = 0.30, *r*_C_ = 0.47, *r*_B_ = 0.16) was the most central care problem cluster across the three centrality indices, followed by “do not know how to clean themselves after using the toilet” (*r*_S_ = 0.30, *r*_C_ = 0.49, *r*_B_ = 0.10), “urinating and defecating in inappropriate places” (*r*_S_ = 0.30, *r*_C_ = 0.47, *r*_B_ = 0.08), “hitting, kicking, pushing, or biting others” (*r*_S_ = 0.26, *r*_C_ = 0.47, *r*_B_ = 0.07), and “cursing or verbal aggression” (*r*_S_ = 0.28, *r*_C_ = 0.43, *r*_B_ = 0.06).

**Figure 2:**
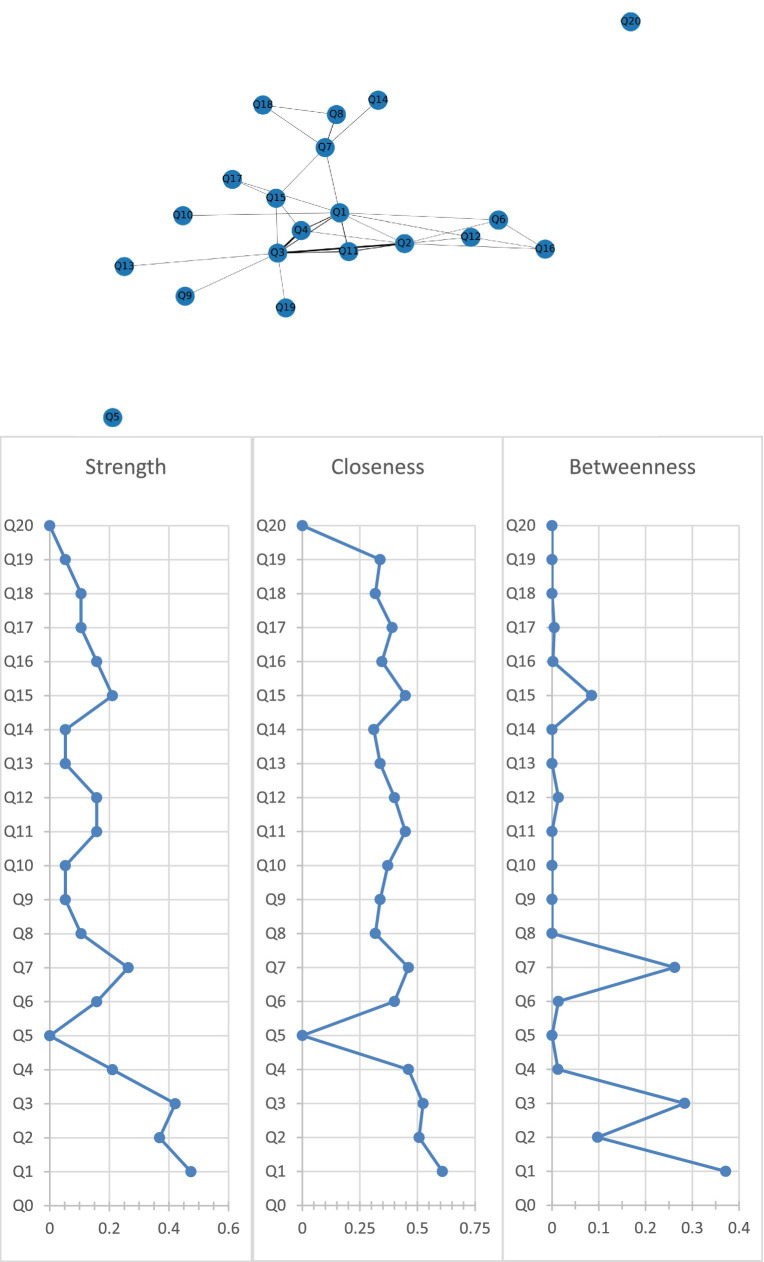
Network of care problem clusters and centrality indices. Q1. Cluster A (Deterioration in activities of daily living); Q2. Cluster B (Paraphasia and psychosis); Q3. Cluster C (Verbal and physical aggression); Q4. Cluster D (Rejection of care); Q5. Cluster E (Safety risks associated with swallowing and walking ability); Q6. Cluster F (Walking function disorder); Q7. Cluster G (Loss of activities of daily living); Q8. Cluster H (Bedridden related complications); Q9. Cluster I (Safety risks related to accidental injury); Q10. Eating or drinking inappropriate substances; Q11. Performing repeated action; Q12. Forgetting that he/she had eaten and wanted to eat again; Q13. Making verbal or physical sexual advances; Q14. Difficulty chewing; Q15. Day and night reversed; Q16. Getting up multiple times during the night; Q17. Having difficulty in falling asleep; Q18. Feeding through a nasogastric tube; Q19. Apathy; Q20. Constipation.

Figure 2 shows the association network and centrality indices among the nine care problem clusters and eleven care problems. The three strongest edges were between Cluster C and Cluster D (*r* = 0.44), Cluster B and Cluster C (*r* = 0.42), and Cluster C and “performing repeated action” (*r* = 0.38). In the entire network, Cluster A (*r*_S_ = 0.47, *r*_C_ = 0.61, *r*_B_ = 0.37) was the most central care problem cluster across the three centrality indices, followed by Cluster C (*r*_S_ = 0.42, *r*_C_ = 0.52, *r*_B_ = 0.28), and Cluster G (*r*_S_ = 0.26, *r*_C_ = 0.46, *r*_B_ = 0.26).

### Regression analysis

The results of the exploratory multiple linear regression models of overall care problems and top three central care problem clusters are shown in Table 3. People with dementia who were single (*β* = −0.074, *p* = 0.041), had longer years of dementia diagnosis (*β* = 0.195, *p* < 0.01), took more types of dementia medication (*β* = 0.163, *p* < 0.01), and dementia caregivers who had lower education level (*β* = −0.089, *p* = 0.011) were significantly associated with more care problems. Variables including people with dementia’s educational attainment, marital status, years of dementia diagnosis, number of dementia medication type, number of chronic diseases, dementia caregivers’ educational attainment, and the relationship between people with dementia and caregivers were significantly associated with the prevalence of these care problem clusters.

**Table 3:** Linear regression of care problems and clusters (*N* = 1,012).

Variable	Model 1 Overall		Model 2 Cluster A		Model 3 Cluster C		Model 4 Cluster G	
	*β*	*p*	*β*	*p*	*β*	*p*	*β*	*p*
Gender (reference = male)	−0.012	0.710	−0.006	0.840	−0.009	0.777	−0.016	0.608
Age	−0.031	0.424	−0.035	0.357	−0.033	0.396	0.048	0.203
Educational attainment	−0.002	0.959	−0.017	0.595	−0.029	0.375	0.091	0.004^**^
Marital status (reference = single)	−0.074	0.041^*^	−0.013	0.713	−0.083	0.023^*^	0.030	0.395
*Diagnosis (reference = other)*								
Alzheimer’s disease	−0.001	0.984	0.065	0.248	−0.020	0.723	−0.070	0.202
Vascular dementia	−0.001	0.981	−0.009	0.844	0.029	0.531	−0.069	0.118
Mixed dementia	0.079	0.099	0.039	0.419	0.093	0.055	0.073	0.119
Years of dementia diagnosis	0.195	<0.001^**^	0.251	<0.001^**^	0.115	<0.001^**^	0.223	<0.001^**^
Number of dementia medication type	0.163	<0.001^**^	0.014	0.670	0.082	0.011^*^	0.096	0.002^**^
Number of chronic disease	0.024	0.445	−0.019	0.546	−0.007	0.824	0.094	0.003^**^
Gender—caregiver (reference = male)	0.033	0.298	0.043	0.175	−0.010	0.765	0.006	0.857
Age—caregiver	0.051	0.328	0.006	0.912	0.068	0.195	0.002	0.963
Educational attainment—caregiver	−0.089	0.011^*^	−0.081	0.022^*^	−0.119	0.001^**^	−0.016	0.634
*Relationship with care recipients (reference = other)*								
Spouse	−0.002	0.980	0.077	0.215	0.033	0.602	−0.030	0.617
Daughter/son	−0.007	0.896	0.071	0.178	0.055	0.302	−0.026	0.617
Daughter/son-in-law	0.032	0.422	0.089	0.024^*^	0.058	0.146	0.003	0.930
*Employment status (reference = unemployed)*								
Full-time job	−0.002	0.968	0.070	0.188	−0.045	0.400	−0.094	0.069
Part-time job	−0.015	0.706	−0.004	0.921	−0.045	0.246	0.070	0.067
Retired	−0.043	0.457	0.060	0.283	−0.033	0.572	−0.020	0.723

^*^p < 0.05, ^**^p < 0.01. Model 1: *F* = 5.163, *p* < 0.001, *R*^2^(adj) = 0.073; Model 2: *F* = 4.840, *p* < 0.001, *R*^2^(adj) = 0.067; Model 3: *F* = 3.935, *p* < 0.001, *R*^2^(adj) = 0.052; Model 4: *F* = 7.556, *p* < 0.001, *R*^2^(adj) = 0.110.

## Discussion

This study aimed to identify care problem clusters and core care problems, and explore demographic variables associated with these care problem clusters among caregivers of people living with dementia. Nine care problem clusters were identified, including “Deterioration in activities of daily living,” “Paraphasia and psychosis,” “Verbal and physical aggression,” “Rejection of care,” “Safety risks associated with swallowing and walking ability,” “Walking function disorder,” “Loss of activities of daily living,” “Bedridden related complications,” and “Safety risks related to accidental injury.” In the entire care problem network, “Deterioration in activities of daily living” was the most core care problem cluster across the three centrality indices, followed by “Verbal and nonverbal aggression” and “Loss of activities of daily living.” Variables including marital status, years of dementia diagnosis, number of dementia medication type, and caregiver’s educational attainment were associated with the prevalence of these three care problem clusters.

A shift in research interest from separate care problem to care problem clusters among caregivers of people living with dementia may contribute to a better understanding of dementia care, as related care problem clusters may respond to the same nursing or treatment interventions (37–39). Nine care problem clusters were derived from the data in this study. A previous study (38) showed that BPSD were not independent, but consist of a group or cluster of related symptoms, which was consistent with our findings. Another previous study revealed the co-occurrence of symptoms between hallucinations and delusions, and hinted at a common etiology and treatment for these symptoms (40). In our study, the “Paraphasia and psychosis” cluster (Cluster B) also contained hallucinations and delusions, suggesting that the care problems in the cluster may be treated as a whole to optimize care and treatment plan. Some researchers proposed that BPSD was not a unitary concept, instead it should be divided into several symptom groups, each of which may reflect different psychosocial determinants, biological correlates, and disease duration (41), which was in line with our research philosophy. However, the above studies only focused on the presence of BPSD, while our study focused not only on BPSD, but also on a series of care problems related to daily life and safety risks. A recent study (21) conducted cluster analysis based on the minimum spanning tree algorithm on 687 dementia samples and obtained 7 care problem clusters, which was not completely consistent with our research results. The reasons for the difference in results may be different sample sizes and analysis methods. In terms of sample size, our study was based on a large sample of 1,012 cases; in terms of analysis methods, the minimum spanning tree only focused on the strength between care problems, while the network analysis used in our study focused not only on the strength, but also on the betweenness and closeness between care problems.

Our study demonstrated that “Deterioration in activities of daily living” cluster (Cluster A) was the most core care problem cluster across the three centrality indices in the entire care problem network. In this cluster, there are mainly some manifestations of functional degradation, such as “Do not know how to dress in order,” “Do not know how to clean themselves after using the toilet,” and “Do not know how to use tableware properly.” Studies (42, 43) showed that functional decline in activities of daily living appeared more pronounced and disrupted more aspects of life activities for individuals with dementia versus individuals without dementia. Our study also found that “Loss of activities of daily living” cluster (Cluster G) was the third core care problem cluster across the three centrality indices. This cluster includes “Losing the ability to communicate,” “Bedridden,” and “Urinary and fecal incontinence.” Dementia is a progressive disease. As the disease progresses, it may eventually lead to loss of multiple functions, which will bring greater burden to caregivers. The deterioration of activities of daily living is common in people living with dementia, which suggests that nursing interventions aimed at delaying the functional deterioration may need to be prioritized in order to optimize the care plan and improve the quality of life of people living with dementia.

Similar to “Deterioration in activities of daily living” cluster (Cluster A), we found high coefficients of the three centrality indices in “Verbal and physical aggression” cluster (Cluster C) among all included care problems. In this cluster, verbal aggression includes “Cursing,” “Screaming,” and “Making strange noises—such as laughs, crying,” and physical aggression includes “Hitting, kicking, pushing, or biting others” and “Throwing things, tearing things, or destroying property.” Verbal and physical aggressive behaviors in people living with dementia have been shown to be highly prevalent in many previous studies (44–46). In addition, some studies demonstrated that verbal aggression and physical aggression often coexist (47, 48). Our results were in line with the findings from these studies. Mechanisms behind the co-occurrence of verbal aggression and physical aggression are multifactorial, including different neurobiological factors as well as social, psychological, and environmental factors. The onset of physical aggression in dementia patients can be very dangerous for caregivers because it is often an unexpected attack. The above information suggests that for dementia patients with verbal aggression, caregivers should strengthen observation, raise vigilance, find out the potential causes of verbal aggression and actively carry out nursing intervention, so as to prevent the occurrence of physical aggression and avoid harm to caregivers.

Our study revealed that the strongest edge was between “Verbal and physical aggression” cluster (Cluster C) and “Rejection of care” cluster (Cluster D) (*r* = 0.44), followed by “Paraphasia and psychosis” cluster (Cluster B) and “Verbal and physical aggression” cluster (Cluster C) (*r* = 0.42) among nine care problem clusters. A higher strong edge means that the symptom cluster is more likely to occur in conjunction with other symptom clusters. “Rejection of care” cluster (Cluster D) covers care problems such as “Refusing to take a bath,” “Refusing to wear clothes,” “Refusing to eat or refusing to be fed” and other manifestations of refusal to care. In the practice of caring for people living with dementia, we often find that if the caregiver forces the people living with dementia to do something he does not want to do, such as taking a shower, it is likely to induce verbal aggression or physical aggression (49, 50). This practical experience also indirectly corresponds to the strong correlation between “Rejection of care” cluster (Cluster D) and “Verbal and physical aggression” cluster (Cluster C). In “Paraphasia and psychosis” cluster (Cluster B), the main care problems included hallucinations, delusions, etc. Dementia patients with these care problems are prone to delusions that distort facts, such as firmly believing that others have stolen their money, and easily triggering verbal or physical aggression when others argue with them. From this perspective, the strong correlation between “Paraphasia and psychosis” cluster (Cluster B) and “Verbal and physical aggression” cluster (Cluster C) is well explained. The above reminds us that in the process of caring for people living with dementia, we should follow his will and recognize his ideas, even if his ideas are wrong, so as to avoid inducing his aggressive behavior.

The sociodemographic characteristics of dementia patients and their caregivers may have an impact on the occurrence of care problems. This study revealed that marital status, years of dementia diagnosis, number of dementia medication type, and caregiver’s educational attainment were associated with the prevalence of overall care problem clusters. In terms of marital status, being married was a protective factor against having “Verbal and physical aggression” cluster (Cluster C). The possible reason is that the familiarity and intimacy between dementia patients and their spouse caregivers can give them enough sense of security, avoid intergenerational conflicts, and therefore reduce the occurrence of aggressive behavior (51, 52). In terms of disease course, the longer the dementia was diagnosed, the more likely to have “Deterioration in activities of daily living” (Cluster A), “Verbal and physical aggression” cluster (Cluster C), and “Loss of activities of daily living” cluster (Cluster G). In other words, as the disease progresses, people with dementia gradually deteriorate and lose various functions, and are more likely to develop BPSD. Rockwood et al. (53) used two analytical methods (connectivity graph analysis and multiple correspondence analysis) to identify psychotic symptom clusters and found that moderate/severe dementia was associated with more psychotic symptoms, which was consistent with our results. Mouriz-Corbelle et al. (54) and Umesh et al. (55) also found similar results that high level of aggression was associated with low level of cognitive function.

In terms of number of dementia medication type, the more types of medication taken by dementia patients, the more likely “Verbal and physical aggression” cluster (Cluster C) and “Loss of activities of daily living” cluster (Cluster G) were induced. This finding was consistent with the results of a recent study that showed that exacerbated BPSD were associated with patients taking psychotropic drugs (56). In addition to dementia medications, we should also consider that this population may be taking medications for other age-related diseases (such as hypertension, diabetes, hypercholesterolemia), which may increase the risk of adverse interactions with their dementia medications. This finding indicates that excessive use of dementia medications may cause serious side effects or adverse interactions with medications for other age-related diseases, so it is important to weigh the benefits against the risks before taking them. In addition, our study found that higher education level of caregivers had a protective effect on the occurrence of “Deterioration in activities of daily living” (Cluster A) and “Verbal and physical aggression” cluster (Cluster C), which may be that caregivers with higher education level had stronger ability to acquire dementia care knowledge, so the quality of care for patients is higher (57, 58). This result suggests that we should strengthen guidance and support for caregivers with low education level to enhance their knowledge reserve and improve care outcomes.

### Strengths and limitations

This is the first time that the network analysis has been applied to explore the care problem clusters and core care problems among caregivers of people living with dementia. Network analysis has sufficient statistical power to identify care problem clusters and core care problems through three centrality indicators, namely, strength, closeness, and betweenness. In addition, compared with other studies, the care problems that we focused on are more comprehensive. Our study focused not only on BPSD, but also on a series of care problems related to daily life and safety risks. What’s more, our study not only identified the core care problem cluster, but also explored the influencing factors, which can provide guidance for dementia care. This study also has several limitations that should be recognized. First, we used convenient sampling method, and the survey data were collected mainly from caregivers of dementia patients in Beijing, Tianjin, Guangzhou, and Hangzhou. Due to the limited sample representativeness, our findings cannot be generalized to the entire Chinese dementia population. Second, only a cross-sectional design was used, and care problems were not tracked longitudinally, which may be useful to examine changes in the prevalence of care problems over time. Last, additional subsample analysis by disease severity and type was not performed. With the accumulation of sample size, care problem clusters with different dementia severity and dementia types can be further identified in the future.

## Conclusion

Our study generated new knowledge of symptom network among people living with dementia by identifying nine care problem clusters. In the entire care problem network, “Deterioration in activities of daily living” was the most core care problem cluster, followed by “Verbal and nonverbal aggression” cluster. Our study suggests that there is a need to evaluate care problem clusters for the improvement of care problem management among people living with dementia. Caregivers need to consider each care problem in its own right and also to be aware of the interrelations between them when assessing patients and developing strategies for care. It is particularly important to include assessment and treatment of core care problem as an essential component of the dementia care.

## Data availability statement

The raw data supporting the conclusions of this article will be made available by the authors, without undue reservation.

## Ethics statement

The studies involving human participants were reviewed and approved by the Peking University Biomedical Ethics Committee (IRB00001052-21095). The patients/participants provided their informed consent to participate in this study.

## Author contributions

SH and ZZ participated in the research design. ML and SH drafted the manuscript. ML, YaZ, and YS contributed the acquisition of data. YiZ and XY performed the data analysis. ZW reviewed and edited the manuscript. All authors contributed to the article and approved the submitted version.

## Funding

This work was supported by the National Natural Science Foundation of China (grant number 72274007) and the China Postdoctoral Science Foundation (grant number 2023M732119).

## Conflict of interest

The authors declare that the research was conducted in the absence of any commercial or financial relationships that could be construed as a potential conflict of interest.

## Publisher’s note

All claims expressed in this article are solely those of the authors and do not necessarily represent those of their affiliated organizations, or those of the publisher, the editors and the reviewers. Any product that may be evaluated in this article, or claim that may be made by its manufacturer, is not guaranteed or endorsed by the publisher.
