# Supplementary material for: Identifying care problem clusters and core care problems of older adults with dementia for caregivers: a network analysis
Source: Front Public Health. 2023 Aug 10;11:1195637. doi: 10.3389/fpubh.2023.1195637 (PMC10449331; doi:10.3389/fpubh.2023.1195637)
Supplement: Supplementary file 1 [file Table_1.DOCX]

**Dementia caregiver’s care problem checklist**

| **Daily living care problems** (What are the following issues that have troubled you in the past 2 weeks?) | No | Yes |
| --- | --- | --- |
| **Eating** | | |
| 1. Forgetting that he/she had eaten and wanted to eat again |  |  |
| 2. Do not know how to choose food |  |  |
| 3. Do not know how to use tableware properly |  |  |
| 4. Eating or drinking inappropriate substances |  |  |
| 5. Refusing to eat or refusing to be fed |  |  |
| 6. Difficulty chewing |  |  |
| 7. Dysphagia |  |  |
| 8. Feeding through a nasogastric tube |  |  |
| **Dressing** | | |
| 9. Cannot choose clothes that suit the season |  |  |
| 10. Do not know how to dress in order |  |  |
| 11. Wearing the same clothes and refusing to change |  |  |
| 12. Inappropriate dressing or disrobing |  |  |
| 13. Refusing to wear clothes |  |  |
| **Excreting** | | |
| 14. Cannot find the toilet on his/her own |  |  |
| 15. Do not know how to use the toilet |  |  |
| 16. Do not know how to clean themselves after using the toilet |  |  |
| 17. Urinating and defecating in inappropriate places |  |  |
| 18. Urinary and fecal incontinence |  |  |
| 19. Constipation |  |  |
| **Bathing and washing** | | |
| 20. Refusing to take a bath |  |  |
| 21. Forgetting steps to wash or brush |  |  |
| 22. Refusing to freshen up |  |  |
| 23. Aggressive behavior when assisting in bathing |  |  |
| **Walking** | | |
| 24. Bedridden |  |  |
| **Sleeping** | | |
| 25. Having difficulty in falling asleep |  |  |
| 26. Getting up multiple times during the night |  |  |
| 27. Day and night reversed |  |  |
| **Communication** | | |
| 28. Having difficulty in understanding what others are saying |  |  |
| 29. Having difficulty in expressing his/her own thoughts clearly |  |  |
| 30. Losing the ability to communicate and can only repeat simple words |  |  |
| **Behavioral and psychological problems** (What are the following issues that have troubled you in the past 2 weeks?) | No | Yes |
| 31. Cursing or verbal aggression |  |  |
| 32. Hitting, kicking, pushing, or biting others |  |  |
| 33. Throwing things, tearing things, or destroying property |  |  |
| 34. Making verbal or physical sexual advances |  |  |
| 35. Pacing and aimless wandering |  |  |
| 36. Performing repeated action |  |  |
| 37. Saying the same thing or asking the same question repeatedly |  |  |
| 38. Hiding valuable things, or hoarding worthless things |  |  |
| 39. Constantly requesting help or attention |  |  |
| 40. Complaining |  |  |
| 41. Making strange noises–such as laughs, crying |  |  |
| 42. Screaming |  |  |
| 43. Hallucination |  |  |
| 44. Delusion |  |  |
| 45. Apathy |  |  |
| **Safety risk problems** (What are the following safety risks that have troubled you in the past 3 months?) | No | Yes |
| 46. Falls |  |  |
| 47. Falling out of bed |  |  |
| 48. Self-injury |  |  |
| 49. Hurting others |  |  |
| 50. Sneaking out |  |  |
| 51. Getting lost |  |  |
| 52. Accidental aspiration |  |  |
| 53. Eating or drinking inappropriate substances |  |  |
| 54. Irritating cough |  |  |
| 55. Choking on food |  |  |
| 56. Pneumonia |  |  |
| 57. Infection |  |  |
| 58. Pressure injury |  |  |
